# The miR151 and miR5100 Transfected Bone Marrow Stromal Cells Increase Myoblast Fusion in IGFBP2 Dependent Manner

**DOI:** 10.1007/s12015-022-10350-y

**Published:** 2022-02-21

**Authors:** Bartosz Mierzejewski, Zuzanna Michalska, Daniel Jackowski, Władysława Streminska, Katarzyna Janczyk-Ilach, Marta Koblowska, Roksana Iwanicka-Nowicka, Agnieszka Gromadka, Maria Anna Ciemerych, Edyta Brzoska

**Affiliations:** 1 Department of Cytology, Faculty of Biology, University of Warsaw, Miecznikowa 1 St, 02-096 Warszawa, Poland; 2 Laboratory of Systems Biology, Faculty of Biology, University of Warsaw, 02-096 Warsaw, Poland; 3 Laboratory of Microarray Analysis, Institute of Biochemistry and Biophysics, Polish Academy of Sciences, 02-106 Warsaw, Poland; 4 Department of Bioinformatics, Institute of Biochemistry and Biophysics, Polish Academy of Sciences, 02-106 Warsaw, Poland

**Keywords:** bone marrow-derived mesenchymal stem/stromal cells, myoblasts, SDF-1, miR151, miR5100, migration, fusion, proliferation

## Abstract

**Background:**

Bone marrow stromal cells (BMSCs) form a perivascular cell population in the bone marrow. These cells do not present naïve myogenic potential. However, their myogenic identity could be induced experimentally *in vitro* or *in vivo*. *In vivo*, after transplantation into injured muscle, BMSCs rarely fused with myofibers. However, BMSC participation in myofiber reconstruction increased if they were modified by *NICD* or *PAX3* overexpression. Nevertheless, BMSCs paracrine function could play a positive role in skeletal muscle regeneration. Previously, we showed that SDF-1 treatment and coculture with myofibers increased BMSC ability to reconstruct myofibers. We also noticed that SDF-1 treatment changed selected miRNAs expression, including miR151 and miR5100.

**Methods:**

Mouse BMSCs were transfected with miR151 and miR5100 mimics and their proliferation, myogenic differentiation, and fusion with myoblasts were analyzed.

**Results:**

We showed that miR151 and miR5100 played an important role in the regulation of BMSC proliferation and migration. Moreover, the presence of miR151 and miR5100 transfected BMSCs in co-cultures with human myoblasts increased their fusion. This effect was achieved in an IGFBP2 dependent manner.

**Conclusions:**

Mouse BMSCs did not present naïve myogenic potential but secreted proteins could impact myogenic cell differentiation. miR151 and miR5100 transfection changed BMSC migration and IGFBP2 and MMP12 expression in BMSCs. miR151 and miR5100 transfected BMSCs increased myoblast fusion *in vitro*.

**Graphical abstract:**

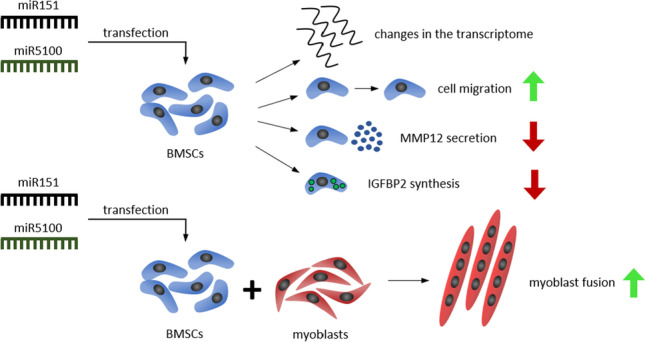

**Supplementary Information:**

The online version contains supplementary material available at 10.1007/s12015-022-10350-y.

## Background

Bone marrow stromal cells (BMSCs) form a heterogeneous perivascular cell population in bone marrow [1]. They present the ability to participate in bone and bone marrow reconstruction and create the hematopoietic stem cell niche [2, 3]. They include a population of cells that fulfil the rigorous criteria defining stem cells, such as the ability to self-renew and differentiate *in vivo,* at single cell level [4–8]. Thus, BMSCs have osteogenic, adipogenic, and chondrogenic potential. Their potential to fuse or differentiate into myoblasts is not profound [9]. However, it could be induced with low efficiency. Rat BMSCs could follow myogenic differentiation, i.e., undergo myotube formation, in response to DNA demethylating drug—5-azacytidine [10]. Human BMSCs do not form myotubes in the absence of myoblasts but infrequently fuse with such cells when cultured in differentiation inducing medium [2, 11]. Moreover, overexpression of *NICD* and culture of human BMSCs in the medium supporting differentiation led to the formation of cells expressing *PAX7*, *MYOD, myogenin,* and resulted in myotube formation [12]. Similar effect was observed as a result of overexpression of *Ctnnb1* encoding β-catenin or *Pax3* in rat, mouse, or human BMSCs [13–15]. Next, the interaction of BMSCs with myofibers induced the expression of myogenic regulatory factors (MRFs) [16]. However, after transplantation into injured muscles, BMSCs were rarely able to fuse and form myofibers [9, 17]. Nevertheless, all of the mentioned methods of BMSC myogenic identity induction improved their efficiency to participate in myofiber reconstruction *in vivo* [10–16].

The most important aspects of transplanted cells participation in skeletal muscle reconstruction are their ability to migrate, undergo myogenic differentiation, as well as their paracrine function. Cell migration is crucial for the efficiency of tissue engraftment [18–20]. There are numerous chemokines that induce cell migration upon interaction with their cognate receptors [21]. One of them is stromal derived factor – 1 (SDF-1), i.e., CXCL12 [21]. Our previous study showed that SDF-1 increased BMSCs migration and the ability to reconstruct myofibers [16]. SDF-1 binds two receptors, i.e., CXCR4 and CXCR7 (ACKR3) [17, 22]. CXCR4 acts through G protein which subunits activates phosphoinositide 3-kinase (PI3K)/AKT, phospholipase C, and extracellular signal-regulated kinase 1/2 (ERK1/2), i.e., MAP kinase (MAPK) pathways. CXCR7 acts through β-arrestin and induces pathways, such as protein kinase B (AKT), ERK1/2 MAPK, and Janus kinase (JAK)/signal transducer and activator of transcription 3 (STAT3). As many other cytokines, also SDF-1 action leads to miRNA changes, however, little is known about such interconnections.

miRNAs are small (~22 nucleotides long) non-coding RNAs which are involved in post-transcriptional regulation of gene expression. miRNAs can bind to a 3’ untranslated region (3’-UTR) of target mRNA resulting in either their degradation or inhibition of protein translation [23, 24]. They are known to regulate many different biological processes and play an important roles in cell proliferation, migration, and differentiation [25]. For example, miR141 was shown to inhibit rat BMSC proliferation *in vitro* by direct targeting of SRY-Box Transcription factor 11 (SOX11) [26]. miR19b-3p overexpression induces human BMSC proliferation and expression of osteogenic differentiation related proteins: Runt-related transcription factor 2 (RUNX2), collagen type 1, alpha 1 (COL1), and alkaline phosphatase (ALP) [26]. miR335-5p promotes osteogenic differentiation by regulating Dickkopf-related protein 1 (DKK1), a known inhibitor of WNT signaling and osteogenic differentiation [27]. Furthermore, some miRNAs were shown to regulate the switch between osteogenic and adipogenic differentiation of BMSCs. miR130a and miR149-3p induce osteogenic and attenuate adipogenic differentiation of BMSCs, while miR188 was described as a key regulator of age-related switch to adipogenesis in these cells [28–30]. Other miRNAs can impact the BMSC migration abilities through AKT-related pathways. miR31 was shown to promote CXCR4/AKT axis and have beneficial effects on BMSC survival and migration [31]. Similarly, miR21 activates PI3K/AKT pathway and promotes BMSC migration by upregulation of metalloproteinase 2 (MMP2) and MMP9 expression (32). Furthermore, miR200a induces the expression of MMPs, especially MMP3 and MMP13, and therefore BMSC migration. Its action is based on downregulation of forkhead box protein J1 (FOXJ1), what leads to activation and translocation into nucleus of nuclear factor-kappa B (NFκB) and increased expression of MMPs [33].

We showed that SDF-1 treatment changed microRNA profile in stem cells (*manuscript in preparation*) and selected microRNAs possibly involved in cell mobilization, such as miR151 and miR5100. Our present study showed that the miRNA151 and miR5100 level changes in BMSCs treated with SDF-1. We noticed here that these molecules played an important role in the regulation of BMSC proliferation and migration. Moreover, the presence of miR151 and miR5100 transfected BMSCs in co-cultures with myoblasts increased their fusion in IGFBP2 dependent manner.

## Materials and Methods

The animal studies were approved by the Local Ethics Committee No. 1 in Warsaw, Poland (permit number 668/2018).

### Bone Marrow Stromal Cell Isolation and Culture

Bone marrow stromal cells (BMSCs) were obtained from the femurs and tibia bones of 2–3-month-old C57/BL6 male mice. Briefly, mice were sacrificed by cervical dislocation, and the bones were isolated, cleared from surrounding tissues, and placed into PBS. The bone marrow was rinsed out from the bones with PBS and centrifuged twice. Then, the cell pellet was suspended and plated in culture dishes or culture dishes containing cover slides coated with 3% gelatin (Sigma-Aldrich) solution in water, and cultured under standard conditions: 37 °C, 5% CO_2_. Cells were expanded in a medium supporting growth (so-called growth medium) composed of: DMEM with glucose 4.5g/l (ThermoFisher Scientific), 15% FBS (ThermoFisher Scientific), and 1% gentamycin (ThermoFisher Scientific). The medium was replaced every 2 days.

### miRNA Transfection and SDF-1 Treatment

3x10^3^/cm^2^ BMSCs were seeded and cultured using growth medium to obtain at least 50% confluence. One hour before the transfection, the medium was changed to OptiMem (ThermoFisher Scientific) supplemented with 15% FBS. Cells were transfected using Lipofectamine 3000 (ThermoFisher Scientific) and 30nM or 50nM of *mir*Vana miRNA mimics: either miR1 or one of the following ones: miR151, miR5100 (MC12998, MC10019, MC22449, Ambion), according to manufacturer’s protocol. Cells transfected with miR1 mimic were considered as positive control and cells transfected with Negative Control #1 mimic (4464058, Ambion) as negative control. Additionally, cells were transfected with miRIDIAN miRNA mimic transfection control conjugated with the fluorescent dye Dy547 (CP-004500-01-20, Dharmacon) to evaluate the transfection efficiency. Transfection procedure lasted for 24h or 48h and then cells were washed with PBS and cultured further with human skeletal myoblasts (described below) or trypsinized and collected for analysis. Some non-transfected cell cultures were subjected to 48h-long treatment with 100ng/ml of mouse SDF-1 recombinant protein, and then washed with PBS, trypsinized, and collected for further analysis.

### Co-cultures and Hybrid Myotubes

3x10^4^ BMSCs were cultured for 7 days in growth medium and transfected as described above. After 3 days, 8x10^4^ of human skeletal muscle myoblasts (hSkM; A11440, ThermoFisher Scientific) were added to BMSCs. Co-cultures were performed for the next 7 days in a medium supporting myogenic differentiation, i.e., DMEM supplemented with 2% horse serum (HS ThermoFisher Scientific), and 1% gentamycin (ThermoFisher Scientific). Finally, cells were fixed with 3% PFA in PBS for further analysis. Fusion index was calculated as a percentage of human nuclei present in myotubes compared to all human nuclei visible. Three independent experiments were performed, nuclei were counted from 5 random fields of view.

### Microarray Analysis

Total RNA together with microRNA fraction was extracted from transfected and control BMSCs using RNAqueous-Micro Total Isolation Kit (ThermoFisher Scientific) according to the manufacturer’s protocol. Further, integrity of obtained RNA was analyzed with 2100 Bioanalyzer (Agilent Technologies) using RNA 6000 Nano Lab Chip kit (Agilent Technologies). All RNA samples had an integrity number above 8.5. Whole transcriptome analysis was performed by using Clariom S Pico Assay (Thermo Fisher Scientific, Waltham, MA, USA) according to manufacture protocol. Prepared samples were hybridized to a single mouse Clariom S array and incubated for 16 h in the Affymetrix GeneChip Hybridization Oven 645 at 45 °C, 60 rpm. Arrays were stained using an Affymetrix GeneChip Fluidics Station 450, according to the specific fluidics protocol, and scanned with an Affymetrix GeneChip Scanner 3000 7G. Raw intensity CEL files generated by GeneChip™Command Console™ were imported into Transcriptome Analysis Console (TAC) 4.0 (Applied Biosystems). The microarray data was normalized and analyzed using Transcriptome Analysis Console 4.0 following the TAC user guide. Each analysis of variance was performed by one-way ANOVA. To determine the significance of differentially expressed genes (DEGs), a cut-off for the fold change value ±1.5 and p-value < 0.05 was applied. The list of detected differentially expressed transcripts was analyzed by Ingenuity Pathway Analysis (IPA, QIAGEN Inc.) software to identify significant interactions and pathways. All analysis and corresponding plots were executed following the software guide with limiting the IPA database information to molecules and relationships where the information was experimentally observed. The obtained data were analyzed also with and Transcriptome Analysis Console (TAC).

### Quantified Real-time PCR (qRT-PCR)

Total RNA together with miRNA fraction was extracted from BMSCs control and transfected with mimic miRNAs, using RNAqueous-Micro Total Isolation Kit (ThermoFisher Scientific), according to the manufacturer’s protocol. cDNA based on isolated mRNA was synthesized using RevertAid First-Strand cDNA Synthesis Kit (ThermoFisher Scientific), in accordance with manufacturer’s protocol, under the following conditions: 25 °C for 5 min, 42 °C for 90 min, and 70 °C for 5 min. mRNA levels were assessed using quantitative real-time PCR analysis (qRT-PCR) with TaqMan assay (ThermoFisher Scientific) for the following genes: *Ablim1*, *Adamts2*, *Igfbp2*, *Mmp12*,*, Twf1* (Mm01254316_m1, Mm00478620_m1, Mm00492632_m1, Mm00500554_m1, Mm_00725968_s1) The average expression of hypoxanthine phosphoribosyltransferase 1 (*Hprt1*; Mm03024075_m1). The reaction was performed with TaqMan Gene Expression Master Mix (ThermoFisher Scientific) using LightCycler96 (Roche) in following conditions: preincubation 2 min, 50 °C; preincubation 10 min, 95 °C; amplification (40 cycles) 15 s, 95 °C, and 1 min, 60 °C. All reactions were performed in duplicates. The results were analyzed as positive before 32 cycle. Expression levels were calculated with 2-(ΔCt) formula in reference to the relative expression of average expression of *Hprt1*. All reactions were performed in duplicates. Three independent experiments were performed.

### miRNA Expression Assay

Total RNA together with miRNA fraction was extracted from BMSCs - control and transfected with mimic miRNAs. Extraction was done using RNAqueous-Micro Total Isolation Kit (ThermoFisher Scientific), according to manufacturer’s protocol. Reverse transcription was performed with TaqMan MiRNA Reverse Transcription Kit (ThermoFisher Scientific) and TaqMan miRNA assays (ThermoFisher Scientific) under the following conditions: 30 min, 16°C; 30 min 42 °C; 5 min, 85 °C. miRNA levels were assessed using quantitative real-time PCR analysis (qPCR). RT primers and TaqMan probes were used for specific miRNA: miR1 (002222), miR151 (001190), miR5100 (462702_mat), U6 (001973). The average expression of noncoding U6 snRNA was used as a reference for further calculations. The reaction was performed with TaqMan Universal Master Mix II, no UNG (ThermoFisher Scientific) using LightCycler96 (Roche) in following conditions: preincubation 2 min, 50 °C; preincubation 10 min, 95 °C; amplification (40 cycles) 15 s, 95 °C, and 1 min, 60 °C. All reactions were performed in duplicates. Three independent experiments were performed.

### Migration Assay – Scratch Assay

Migration of BMSCs, control or transfected with mimic miRNAs, was analyzed using scratch wound healing assay [34]. Briefly, cells were cultured to obtain 90% confluence. Next, the cells were scratched from the plate using a plastic tip to create the “wound.” The wound healing manifested by the ability of the cells to refill the created gap was observed. Pictures of the “wound” area were taken in three time points: 0h, 6h, and after 24h. The area of scratch was calculated using Fiji [35] and GraphPad Prism. Three independent experiments were performed.

### Immunocytochemistry

BMSCs, control, transfected with mimic miRNAs, or co-cultures of BMSCs with human skeletal muscle myoblasts, were fixed with 3% PFA in PBS. Next, specimens were washed in PBS and permeabilized in 0.05% Triton X100 (Sigma-Aldrich) in PBS. Further, specimens were washed in PBS and incubated in 0.25% glycine (Sigma-Aldrich) in PBS, followed by incubation in 3% bovine serum albumin (Sigma-Aldrich) with 2% donkey serum albumin (Sigma-Aldrich) in PBS. Next, samples were incubated with primary antibodies: anti-skeletal myosin (M7523, Sigma-Aldrich), anti-human nuclear antigen (ab191181, Abcam) diluted 1:100 in 3% BSA with 2% donkey serum in PBS at 4 °C overnight, followed by incubation with appropriate secondary antibodies conjugated with either Alexa Fluor 488 or 594 (anti-mouse, 21203, anti-rabbit 21207; ThermoFisher Scientific) diluted 1:500 in 1.5% BSA in PBS in room temperature for 2 h. Negative controls included secondary antibodies staining. Cell nuclei were visualized with Hoechst 33342 (ThermoFisher Scientific) diluted 1:1000 in PBS. Specimens were mounted with Fluorescent Mounting Medium (Dako Cytomation) and analyzed using confocal microscope LSM 700 (Zeiss) and ZEN software (Zeiss). Three independent experiments were performed.

### Flow Cytometry

BMSCs, control or transfected with mimic miRNAs, were collected by trypsinization, rinsed twice with PBS, and incubated with 3% BSA in PBS at 4°C. Further, cells were labelled with fluorescent-conjugated antibodies to detect the following antigens: CD44-APC (561862; BD Biosciences), CD34-FITC (553733; BD Biosciences), CD146-PE (562196; BD Biosciences). Labelled cells were subjected to flow cytometry analysis (CytoFLEX, Beckman Coulter) using CytExpert software. Unlabeled cells were used as negative control. Three independent experiments were performed.

### Cell Proliferation Assay

BMSCs, control or transfected with mimic miRNAs, were incubated in 0.5 μM carboxyfluorescein succinimidyl ester (CFSE, ThermoFisher) in PBS at 37°C for 10 min. Cells were rinsed in PBS and cultured for 24h or 48h in the growth medium under standard conditions. Next, cells were rinsed in PBS, trypsinized, and subjected to flow cytometry analysis (CytoFLEX, Beckman Coulter) using CytExpert software. Unlabeled cells (negative control) and cells analyzed immediately after labeling with CFSE (positive control) were included into each experiment. Three independent experiments were performed.

### Western Blotting

Proteins were isolated from BMSCs, control or transfected with mimic miRNAs, using RIPA buffer (ThermoFisher) supplemented with protease and phosphatase inhibitors. 25 μg of total protein lysates were denatured by boiling in Laemmli buffer, separated using SDS-Page electrophoresis, and transferred to PVDF membranes (Roche). The membranes were blocked with 5% milk/Tris-buffered saline (TBS) for 1h and incubated with primary antibodies diluted 1:1000 in 5% milk/TBS, at 4°C, overnight, followed by secondary antibodies diluted 1:20000, at room temperature, for 2h. Next, protein bands were visualized with SuperSignal West Dura Extended Duration Substrate (Thermo Scientific) and exposed to chemiluminescence positive film (Amersham Hyperfilm ECL, GE Healthcare). Film was developed in a darkroom using a developer and fixer (Fuji). The density of the examined bands was compared to the density of α-tubulin bands. The following primary antibodies were used: rabbit polyclonal anti-IGFBP2 (Abcam) and mouse monoclonal anti-α-tubulin (Sigma-Aldrich). Secondary antibodies used: peroxidase-conjugated rabbit anti-mouse IgG (Sigma-Aldrich) and peroxidase-conjugate goat anti-rabbit IgG (Sigma-Aldrich). Three independent experiments were performed. The blots were analyzed using Gel Doc XR+ and Image Lab 5.1 (BioRad).

### ELISA Assay

48h after BMSCs transfection with miR151 or miR5100 2 ml of culture medium was collected and frozen. Medium was analyzed using ELISA assay to determine the concentration of MMP12 (ab213878, Abcam). Assay was performed according to the manufacturer’s protocol. 562 nm absorbance was measured using a microplate reader BioTek ELx800 (Agilent) with Gen5 software (Agilent). The values obtained from the medium were excluded from analysis. Three independent experiments were performed in duplicates. The average results for each experiment were shown on graphs.

### Statistical Analysis

The Gaussian distribution of values was analyzed with Shapiro-Wilk normality test. The fold change was calculated by comparing the average values of non-treated samples to those of all samples. The data were analyzed using one-way or two-way ANOVA test and post hoc with Dunnett’s multiple comparisons test. All data was compared to results coming from analyzes of control group, i.e., non-treated cells. The differences were considered statistically significant when p < 0.05 (marked with asterisks, * - p < 0.05; ** - p < 0.005; *** - p < 0.001; **** - p < 0.0001). The mean value and standard deviation were shown in each graph presented. All statistical analyzes were performed using GraphPad 7 software (Prism).

## Results

### The Characterization of BMSCs

**Figure 1:**
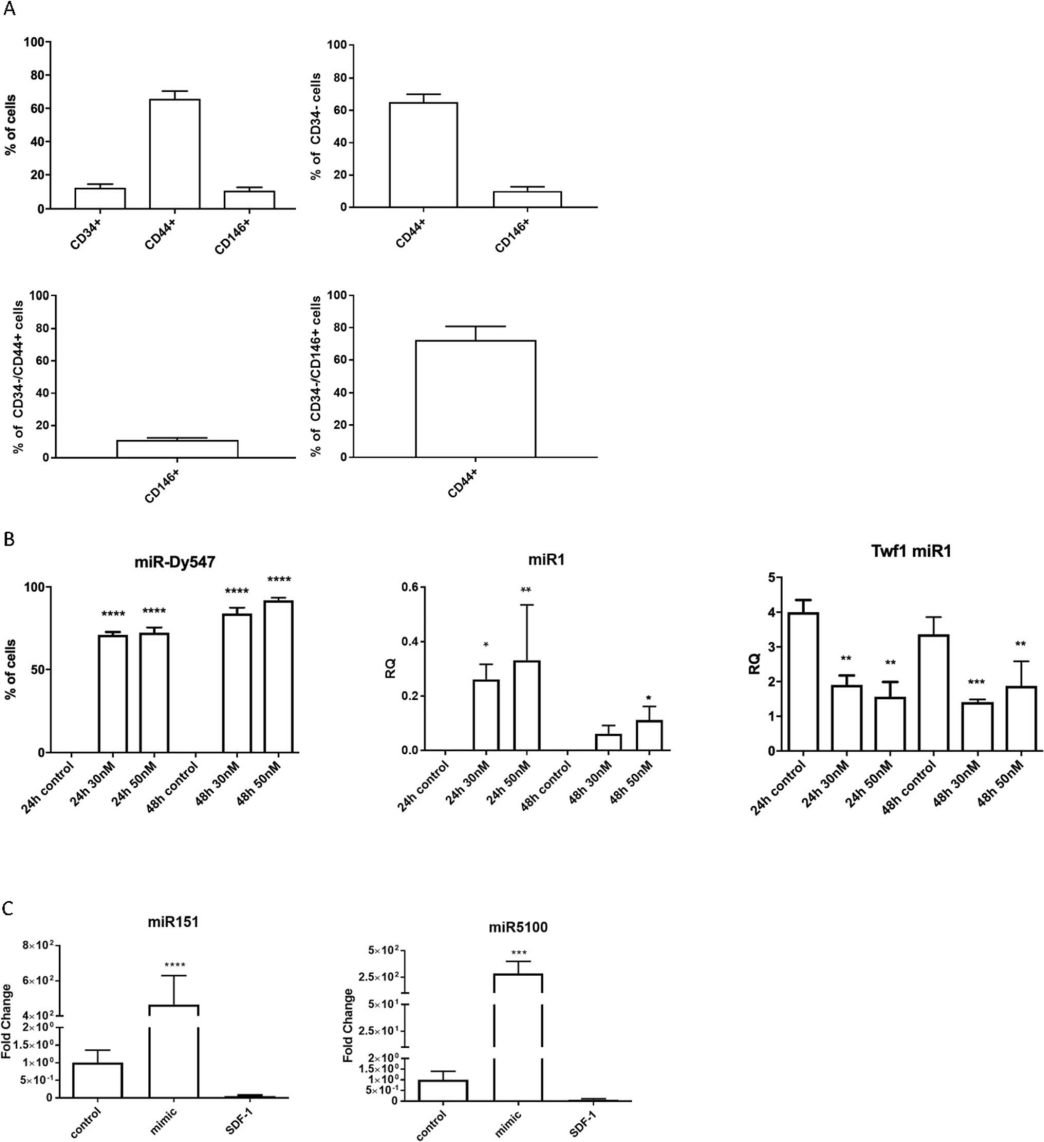
Mouse BMSCs markers and transfection efficiency. (A) Proportion of CD34+, CD44+, and CD146+ cells in the whole cell population obtained from mouse femur and tibia bones. The proportion of CD44+ and CD146+ within CD34- subpopulation. The proportion CD146+ within CD34-/CD44+ subpopulation and CD44+ cells within CD34-/CD146+ subpopulation. (B) Proportion of cells with fluorescent miR-Dy547 present in their cytoplasm after transfection with 30nM or 50nM of miR-Dy547 for 24h or 48h. Expression level of miR1 after transfection with 30nM or 50nM of miR1 mimic for 24h or 48h. Expression levels of miR1 direct target – *Twf1* in control or miR1 mimic transfected mouse BMSCs. (C) Expression levels of miR151 and miR5100 in controls, transfected or treated with SDF-1 mouse BMSCs. The differences were considered statistically significant when p < 0.05 (marked with asterisks, * - p < 0.05; ** - p < 0.005; *** - p < 0.001; **** - p < 0.0001)

BMSCs were isolated from mouse bone marrow and cultured *in vitro* for 7-10 days. Next, the proportion of CD34, CD44, and CD146 positive cells was assessed in *in vitro* cultures (Figure 1A). CD34 is a hematopoietic stem and progenitor cell marker [36]. It is also expressed by other stem and progenitor cells, such as fibro-adipocyte progenitors (FAP), skeletal muscle stem cells, i.e., satellite cells, endothelial and epithelial progenitor cells, and mature endothelial cells [37]. However, CD34 expression in BMSCs is still discussable [36]. CD44 is well-known marker of BMSCs and plays an important role in cell adhesion and homing [37, 38]. CD146 was shown to be expressed by cells that fulfill the criteria of stem cells [3, 39]. In our study, BMSC expressed all selected markers (Fig. 1A, S1). Thus, 12.2% +/- 2.2 of cells expressed CD34, 65.7% +/- 4.6 were CD44+, and 10.5% +/- 2.0 were CD146^+^. The 65.2% +/- 4.9 of CD34^-^ BMSCs expressed CD44^+^ and 10.9% +/- 1.4 of them expressed CD146. The 10.1% +/- 2.7 of CD34^-^CD44^+^ BMSCs expressed CD146^+^ and 72.4% +/- 8.4 of CD34^-^CD146^+^ were CD44^+^. Thus, most of the cells were CD34^-^/CD44^+^/CD146^-^ and small population of BMSCs was CD34^-^/CD44^+^/CD146^+^.

### The Changes in miRNA Level after BMSC Treatment with SDF-1 or miRNA Mimic Transfection

Mouse BMSCs were cultured for 7-10 days and then either treated with SDF-1 or transfected with miRNA mimics used at concentrations 30nM or 50nM. The efficiency of cell transfection was calculated using Dy594 dye-labeled synthetic miRNA that has no identifiable effect on known miRNAs (Figure 1B). The efficiency of transfection was high, i.e., 71.02% +/- 1.7 (30nM miRNA) and 72.3% +/- 3.3 (50nM miRNA) of cells transfected after 24h and 84.0% +/- 3.7 (30nM miRNA) and 92.1% +/- 1.5 (50nM miRNA) after 48h. To better assess the transfection efficiency, we also used mimic miR1 (30nM and 50nM) (Figure 1B). After 24h, the increased level of miR1 was observed, independently of miRNA concentration used. To verify whether miR1 was functional in BMSCs, we tested the expression of *Twf1,* which downregulation depends on this miRNA. The significantly decreased level of *Twf1* expression was noticed 24h and 48h after transfection. We decided to choose 50nM miRNAs and 48h long culture as our experimental setting.

Next, to verify the role of miR151 or miR5100 in mouse BMSC function, these cells were treated with SDF-1 or transfected either with miR151 or miR5100 mimics. Both miRNA molecules were selected based on previous experiments (*manuscript in preparation*) during which we showed that miR151 or miR5100 level was downregulated in SDF-1 treated stem cells and upregulated in those cells in which CXCR4 expression was silenced. First, we followed the output level of miR151 or miR5100 and changes in SDF-1 treated or miR151 or miR5100 mimic transfected BMSCs (Figure 1C). Control cells were neither transfected with mimic miRNAs nor SDF-1 treated. After 48h, miR151 or miR5100 mimics were detected in control BMSCs (Figure 1C). Then, we confirmed our previous observation that miR151 or miR5100 mimic levels decreased in BMSCs 48h after SDF-1 treatment (Figure 1C). Transfection with miR151 or miR5100 mimics significantly increased the level of corresponding miRNAs.

### The Influence of miRNA Mimic Transfection on BMSC Migration and Proliferation and Myogenic Differentiation

**Figure 2:**
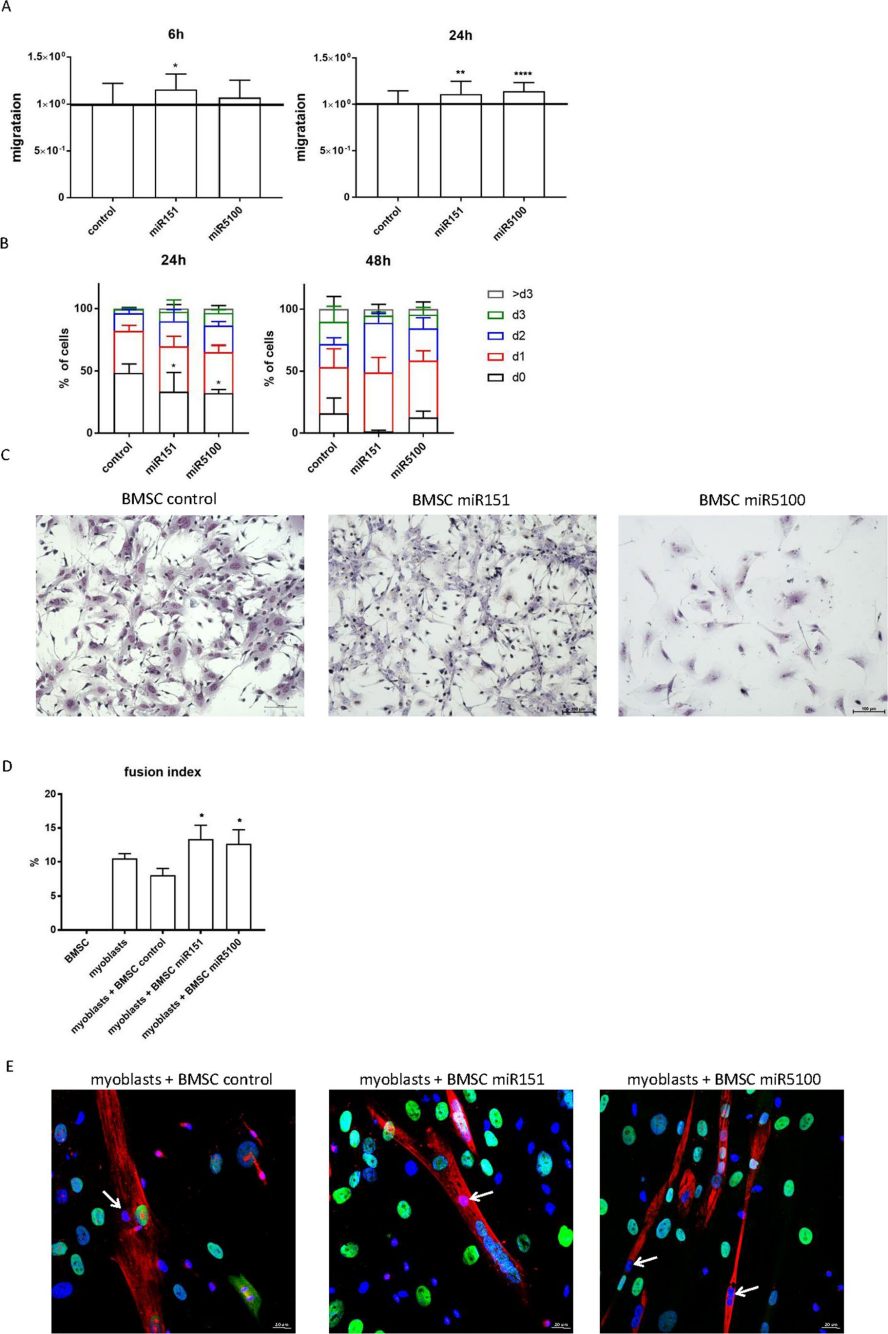
Mouse BMSCs proliferation, migration, morphology, and fusion to human myoblasts. (A) Scratch wound healing assay 6h and 24h after performing the scratch for control or transfected with miR151 or miR5100 BMSCs. (B) Proliferation (CFSE assay) of control or miR151 or miR5100 transfected (24h or 48h) mouse BMSCs. (C) Giemsa staining of control and miR151 or miR5100 transfected mouse BMSCs. (D) Fusion index of human myoblasts cultured in the presence of control or miR151 or miR5100 transfected mouse BMSCs, (E) Visualization of skeletal myosin (red), human nuclear antigen (green) and cell nuclei (blue) in cocultures of myoblasts with control or miR151 or miR5100 transfected BMSCs. Mouse nuclei (blue) within myotubes marked with arrows. Scale: 20 μm. The differences were considered statistically significant when p < 0.05 (marked with asterisks, * - p < 0.05; ** - p < 0.005; *** - p < 0.001)

Since miRNAs selected by us were shown to be downregulated after SDF-1 treatment of stem cells, we checked how their overexpression impacts the migration and proliferation of mouse BMSCs (Figure 2). We expected the decrease of BMSC migration. The cells were transfected with miRNA mimics and after 48h the scratch assay was performed. Migration was assessed 6h and 24h later. Surprisingly, the significantly increased BMSC migration was noticed in case of overexpression of miR151 and miR5100 (Figure 2A). Next, BMSC proliferation was analyzed using CFSE assay. The proportion of not dividing cells significantly decreased 24h after miR151 and miR5100 mimic transfection (Figure 2B). Furthermore, the proportion of cells that underwent two divisions significantly increased 48h post transfection, but only when miR151 mimic was used, compared to control cells. However, neither control BMSCs nor those transfected with miRNA mimics were able to fuse or form multinucleated myotubes alone, i.e., in the absence of exogenous myoblasts (Figure 2C).

Then, mouse BMSCs were co-cultured with human myoblasts in medium stimulating myogenic differentiation (Figure 2D, E). Detection of human nuclei allowed us to distinguish hybrid myotubes, i.e., formed by mouse BMSCs (arrows) and human myoblasts (Figure 2E, green). The fusion index of human myoblasts did not differ in the presence of control mouse BMSCs, compared to human myoblast culture (Figure 2D). Importantly, the presence of miR151 and miR5100 mimic transfected BMSCs increased the fusion of human myoblasts (Figure 2D). Control and miRNA mimics transfected BMSCs rarely fused with human myoblasts and formed hybrid myotubes (Figure 2E). Thus, miR151 and miR5100 mimic transfected BMSCs were able to promote fusion of human myoblasts.

### The Analysis of Changes in the Transcriptome after miRNA Mimic miR151 and miR5100 Transfection

**Figure 3:**
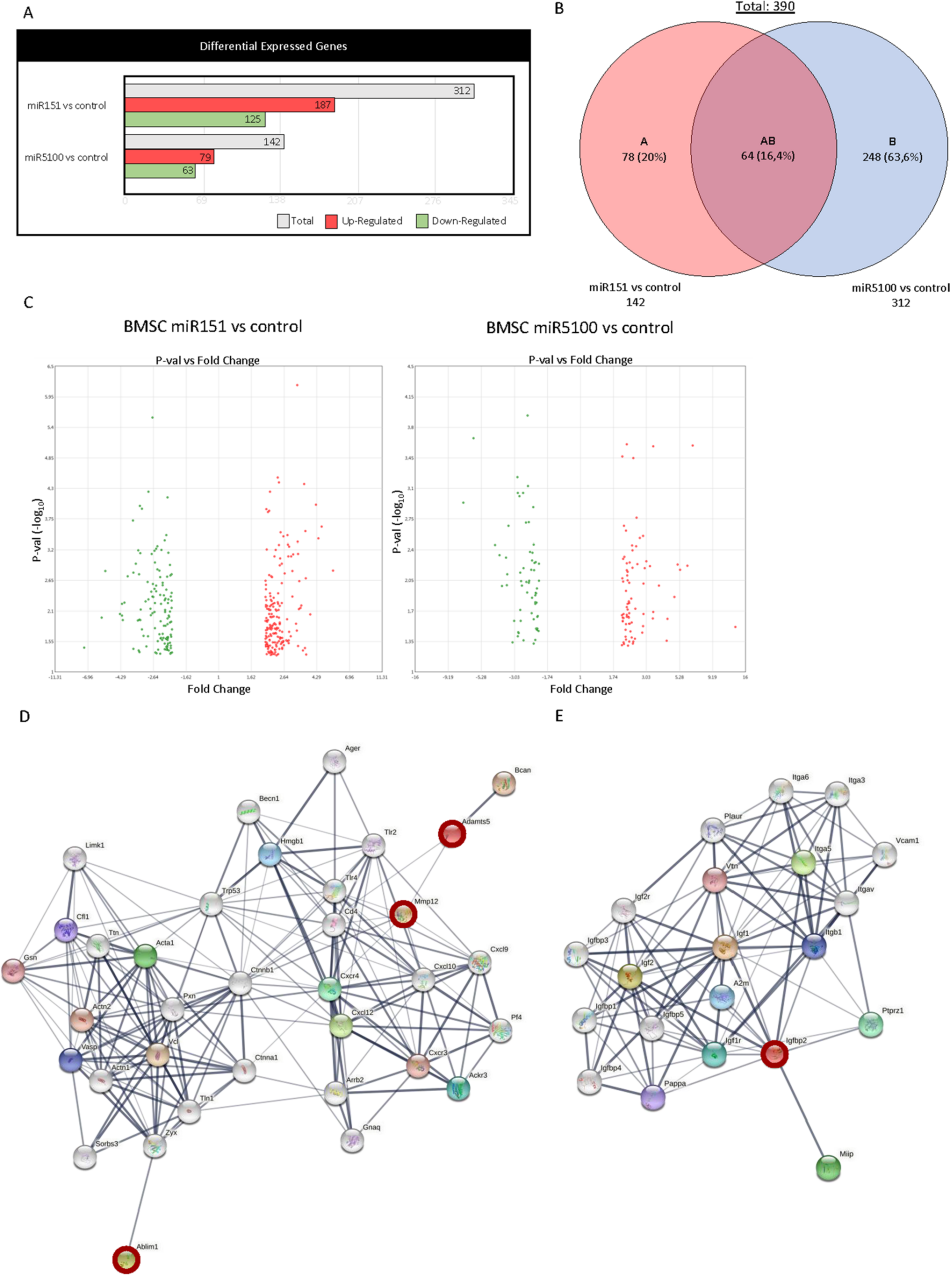
Transcriptome analysis of miR151 or miR5100 transfected mouse BMSCs. (A) The statistically significant changes in gene expression in miR151 or miR5100 transfected mouse BMSCs compared to control, non-transfected cells. Red – up-regulated transcripts; green - down-regulated transcripts; grey – sum of up-regulated and down-regulated transcripts. (B) Venn diagram of differentially expressed genes in miR151 or miR5100 transfected mouse BMSCs compared to non-transfected control cells. (C) Volcano plot of fold changes in gene expression in miR151 or miR5100 transfected mouse BMSCs compared to non-transfected controls. (D) STRING analysis of ABLIM1, ADAMTS5, MMP12, and SDF-1 (CXCL12) interactions. The transcripts characterized by statistically significant difference in expression (microarray analysis) have been highlighted - red. (E) STRING analysis of IGFBP2 interactions. The transcripts characterized by statistically significant difference in expression (microarray analysis) have been highlighted - red.

Considering the impact of miR151 and miR5100 mimic transfection on BMSC migration and fusion, we decided to follow the changes in mouse BMSC transcriptome. Microarray analysis of control cells and those transfected with miRNA mimics miR151 and miR5100 was performed (Figure 3). The Ingenuity Pathway Analysis (IPA) showed that miR151 mimic transfection resulted in statistically significant upregulation of 187 genes and downregulation of 125 genes expression in BMSCs (Figure 3A). The miR5100 mimic overexpression led to statistically significant upregulation of 79 genes and downregulation of 63 genes expression. Interestingly, miR151 influenced the expression of transcripts engaged in the regulation of cellular development, growth, and proliferation. The miR5100 modified the expression of transcripts involved in cellular movement and cell-to-cell signaling and interaction. The Transcriptome Analysis Console (TAC) showed that both molecules, i.e., miR151 and miR5100, statistically significant changed the expression of 64 common transcripts (Figure 3B).

**Figure 4:**
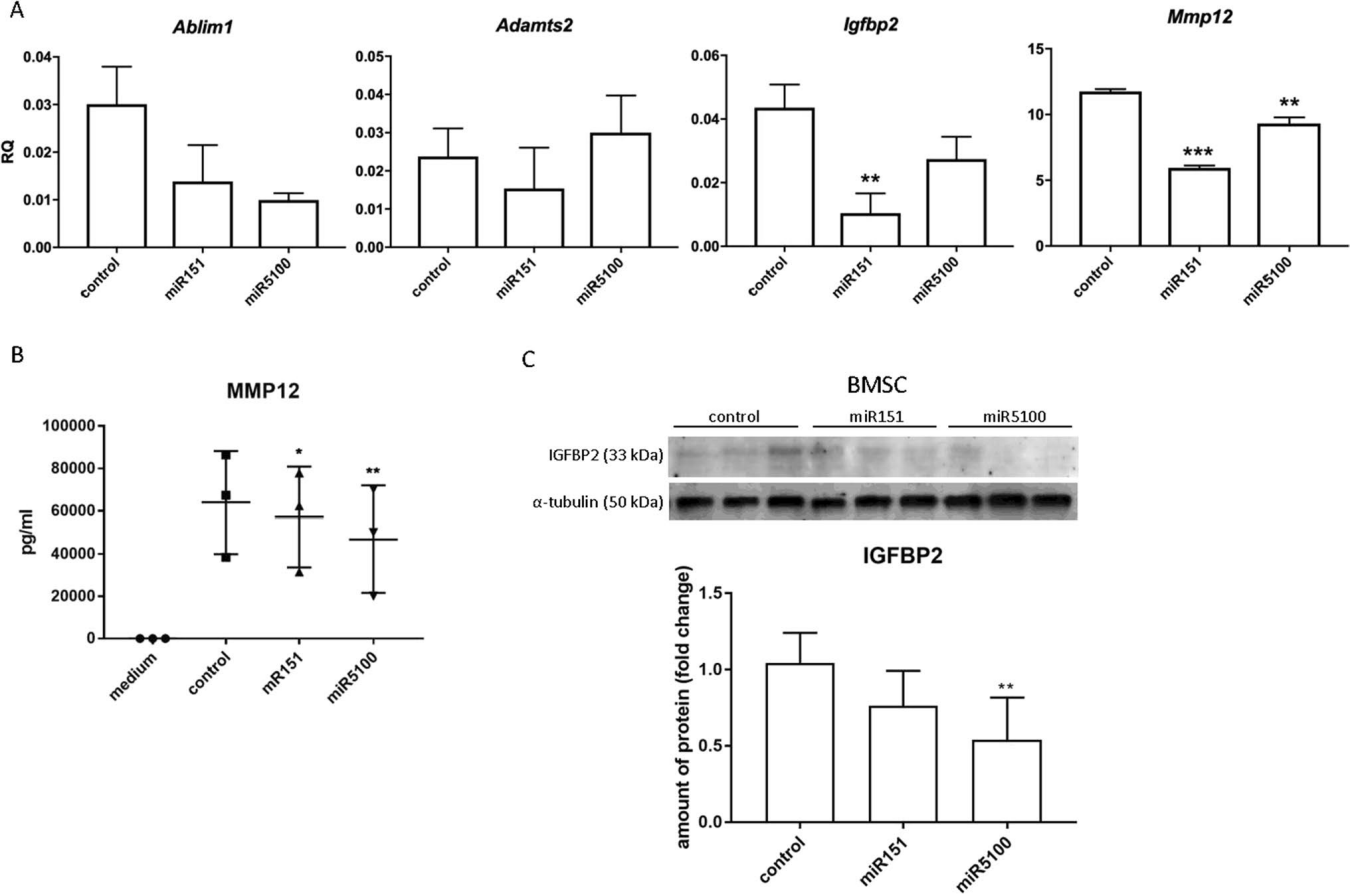
Analysis of selected transcripts and protein levels in miR151 or miR5100 transfected mouse BMSCs. (A) Gene expression levels of selected genes in control or miR151 or miR5100 transfected mouse BMSCs. (B) ELISA analysis of MMP12 amount in medium from *in vitro* cultures of control or transfected mouse BMSCs. (C) The level of IGFBP2 in control or transfected BMSCs. The differences were considered statistically significant when p < 0.05 (marked with asterisks, * - p < 0.05; ** - p < 0.005; *** - p < 0.001)

Our study showed that miR151 and miR5100 impacted mouse BMSC migration and myoblast fusion *in vitro,* in co-culture. We also noticed changes in the level of transcripts engaged in cell migration and fusion, as it was shown by the microarray assay (Figure 3C). STRING analysis was performed for ABLIM1, ADAMTS5, MMP12, and IGFBP2, i.e., genes which changes in expression level were statistically significant in microarray analysis. It showed that selected proteins, i.e., ABLIM1, ADAMTS5, MMP12, and IGFBP2, interplay with each other, participating in the regulation of adhesion, migration, and IGF signaling. Thus, we focused on transcripts engaged in cell migration and fusion, i.e., *Ablim1, Adamts5*, *Mmp12,* and *Igfbp2* that expression was changed in miR151 and miR5100 mimic transfected cells. The changes were confirmed by qRT-PCR analysis (Figure 4A). We noticed significant changes only in the case of *Mmp12* and *Igfbp2* expression. Next, the level of these proteins synthesized by control and miR151 and miR5100 mimic transfected BMSCs was analyzed using ELISA (MMP12) and Western blot (IGFBP2). The level of secreted MMP12 decreased in miR151 and miR5100 mimic transfected BMSCs, compared to control cells (Figure 4B). Importantly, the level of IGFBP2 also seemed to decrease in miR151 and miR5100 mimic transfected BMSCs, however, the differences were statistically significant only between miR5100 transfected cells and control BMSCs (Figure 4C).

## Discussion

The successful outcome of cell transplantation into injured tissues could be limited by many factors. Nevertheless, such treatment may be beneficial. For example, in the case of injured skeletal muscle, transplanted cells could improve regeneration in two ways. First, participating in skeletal muscle reconstruction, second, through paracrine action. Participation in new muscle fibers formation requires effective migration of the transplanted cells within the damaged tissue and their myogenic potential or ability to fuse with myoblasts. Paracrine effects of transplanted cells could, for example, improve endogenous myoblast proliferation, differentiation, fusion, myofiber reconstruction, formation of new neuro-muscular junctions or microvessels, and regulation of inflammation. Cell migration and homing are important both in nonsystemic local transplantation and systemic intra-vascular delivery [20, 40]. Among the factors, which could improve cell migration and skeletal muscle regeneration is SDF-1. The BMSCs do not present naïve myogenic potential [9]. Our previous studies showed that coculture of BMSCs with myofibers and SDF-1 treatment induced their ability to fuse with myoblasts [16]. Moreover, BMSC pretreated with SDF-1 were also able to form new muscle fibers after transplantation [16]. Little is known about SDF-1 interaction with miRNA. Some miRNAs were shown to regulate SDF-1 expression or signaling in many cell types, e.g., zebrafish primordial-germ-cells (miR430), human endothelial cells (miR126), or human BMSCs (miR141-3p) [27, 41, 42]. We observed that SDF-1 treatment changed microRNAs profile in stem cells (*manuscript in preparation*). Thus, we selected microRNAs possibly involved in cell mobilization such as miR151 and miR5100. We hypothesized that they play a role in BMSC migration and/or myogenic identity.

miR151 and miR5100 increased BMSC proliferation and migration. miR151 was described as an important factor regulating cancer cell migration [43–46]. miR151 resides in introne-22 of the host gene encoding focal adhesion kinase (FAK) [43, 46]. The upregulation of miR151 was noticed in human hepatocellular carcinoma cells. It promoted migration *via* downregulation of its target *RhoGDIA* (Rho GDP dissociation inhibitor alpha) expression that led to RAC1, CDC42, and RHO activation [43, 46]. Similarly, in gastric carcinoma cells, the level of miR151 was increased that promoted cell proliferation and migration [47]. Moreover, the expression level of miR151 was correspondingly higher in colon adenocarcinoma tissue compared to normal tissues [48]. On the other hand, miR151 expression was downregulated in other cancer cells and its overexpression decreased proliferation and migration of breast cancer cells in *SOCS5* (suppressor of cytokine signaling 5) dependent manner [44] and of prostate cancer cells through suppression of PI3K/AKT phosphorylation [45]. Moreover, overexpression of miR-151-p increased mouse myoblast proliferation but decreased slow muscle gene expression, i.e., MHC-β/slow and slow muscle troponin I (TnI-S) [49]. Overexpression of miR5100 in lung cancer cells induced their proliferation [50]. It was shown that GTPase RAB6, which is engaged in endocytosis and protein transport, was a direct target of this molecule [50]. miR5100 mediates proliferation and migration of oral squamous carcinoma cells via downregulation of *SCAI* (suppressor of cancer cell invasion) [51]. Thus, both miR151 and miR5100 are involved in the regulation of cancer cell proliferation and migration, however, in a cancer type-dependent manner. Moreover, miR5100 promotes migration and epithelial-mesenchymal transition of lung epithelial cells by targeting TOB2 (transducer of ErbB-2) and its overexpression led to SMAD2/3 activation [52]. By downregulation of *TOB2* expression, miR5100 could also promote osteogenic differentiation of BMSCs [52]. Our study showed that overexpression of miR151 or mR5100 significantly improved mouse BMSC migration and proliferation. In mouse BMSCs we transfected with miR151 or miR5100 mimics, the changes in the level of transcripts engaged, regulation of adhesion, migration, and IGF signaling, were observed.

Both control and miR151 or miR5100 transfected BMSCs did not follow myogenic differentiation in the absence of myoblasts. In cocultures they rarely fused with myoblasts. We previously showed that BMSC fusion with myoblasts is possible - hypoxic condition increased this process [53]. In the present study, we documented that the presence of miR151 or miR5100 transfected mouse BMSCs also increased human myoblast fusion. Importantly, both molecules, i.e., miR151 and miR5100, were previously shown to regulate the level of secreted proteins. miR151 targeted and downregulated its expression in macrophages, what suppressed interleukin 6 (IL6) production [54]. miR151 also impacts IL17 level in endothelial cells [55]. We showed that the level of MMP12 and IGFBP2 was down-regulated in miR151 or miR5100 transfected BMSCs. IGFBPs by binding insulin-like growth factors (IGFs) modulate their availability in microenvironment and control their function and activity [56]. Except regulation of IGFs signaling, IGFBPs act as transcriptional enhancer of *VEGF* gene [56]. It is well established that IGF1 promotes myoblasts proliferation and differentiation [57, 58]. On the other hand, IGFBP2 was recently shown to decrease chicken myoblasts differentiation [59]. Thus, we suggested that the decreased level of IGFBP2 caused by miR151 or miR5100 upregulation in mouse BMSCs could increase the availability of IGF-1. As a result, improved fusion of human myoblasts co-cultured with these cells was improved.

The BMSC secretome could modulate myoblasts differentiation and improve muscle regeneration in many ways [21, 60]. It was documented that during acute or chronic muscle injury and in atrophic muscle, BMSC transplantation impacts skeletal muscle reconstruction and angiogenesis [60]. BMSC transplantation to rat injured muscles improved regeneration and angiogenesis by downregulation of inflammatory cytokines [61]. Transplanted BMSCs also counteracted fibrosis by inhibition of downstream signaling of transforming growth factor β1 (TGFβ1) [61]. BMSCs were also shown to support new muscles formation [62]. Transplanted BMSCs induced skeletal muscle regeneration of mdx mice, a model of Duchenne dystrophy, in SDF-1 dependent manner [63]. However, it should be noticed that BMSCs are not myogenic cells and their transplantation into muscles has limitations such as their naïve potential to differentiate into chondroblasts, osteoblasts and adipoblasts. Thus, overexpression of precisely selected miRNAs in BMSCs could serve as a system to modulate BMSC differentiation, migration, and secretome.

## Conclusions

We showed that two molecules, i.e., miR151 and miR5100, played an important role in the regulation of mouse BMSC proliferation and migration. Moreover, the presence of miR151 and miR5100 transfected mouse BMSCs increased human myoblast fusion in IGFBP2 dependent manner. Importantly, mouse BMSCs did not show naïve myogenic potential, but secreted proteins, such as IGFBP2, could impact myogenic cell differentiation.

## Supplementary Information

Below is the link to the electronic supplementary material.

Supplementary file1 (PDF 262 KB)

## Data Availability

Department of Cytology, Faculty of Biology, University of Warsaw, Miecznikowa 1 St, 02-096 Warsaw, Poland

## Abbreviations

3’-UTR: 3’ untranslated region
ABLIM1: actin binding LIM protein 1
ADAMTS: ADAM metallopeptidase with thrombospondin motif
AKT: protein kinase B
ALP: alkaline phosphatase
BMSC: bone marrow stromal cells
CD: cluster of differentiation
CDC42: cell division cycle 42
CFSE: carboxyfluorescein succinimidyl ester
COL1: collagen type 1, alpha 1
CTNNB1: catenin beta 1
CXCR4: C-X-C Motif chemokine receptor 4
CXCR7: C-X-C Motif chemokine receptor 7
DKK1: Dickkopf-related protein 1
ERK: extracellular signal-regulated kinase
FAK: focal adhesion kinase
FAP: fibro-adipogenic progenitors
FBS: fetal bovine serum
FOXJ3: forehead box protein J 1
GAPDH: glyceraldehyde-3-phosphate dehydrogenase
HPRT1: hypoxanthine phosphoribosyltransferase 1
HS: horse serum
hSkM: human skeletal myoblasts
IGF: insulin growth factor
IGFBP2: insulin growth factor binding protein 2
IL: interleukin
IPA: The Ingenuity Pathway Analysis
JAK: Janus kinase
MAPK: mitogen-activated protein kinase
miRNA: microRNA
MMP: metalloproteinase
MRF: myogenic regulatory factor
MYF5: myogenic factor 5
MYOD: myoblast determination protein 1
NFκB: nuclelar factor-kappa B
NICD: Notch intracellular domain
PAX3: paired-box 3
PAX7: paired box 7
PBS: phosphate buffered saline
PFA: paraformaldehyde
PI3k: phosphoinositide 3-kinase
RAB6: Ras-related protein Rab6
RAC1: Rac family smalol GTPase 1
RHO: rhodopsin
RhoGDIA: Rho GDP dissociation inhibitor, alpha
RUNX2: Runt-related transcription factor 2
SCAI: suppressor of cancer cell invasion
SDF-1/CXCL12: stromal cell-derived factor 1/C-X-C Motif chemokine ligand 12
SMAD: transforming growth factor beta singaling protein
snRNA: small nuclear RNA
SOCS5: suppressor of cytokine signaling 5
SOX11: SRY box transcription factor 11
STAT: signal transduced and activator of transcription proteins
TBS: Tris-buffered saline
TGFβ1: transforming tumor growth factor beta 1
TOB2: transducer of ErbB-2 2
TWF1: twinfilin 1
VEGF: vascular endothelial growth factor
WNT: wingless integrated
