# Supplementary figures and images for: The miR151 and miR5100 Transfected Bone Marrow Stromal Cells Increase Myoblast Fusion in IGFBP2 Dependent Manner
Source: Stem Cell Rev Rep. 2022 Feb 21;18(6):2164–78. doi: 10.1007/s12015-022-10350-y (PMC9391248; doi:10.1007/s12015-022-10350-y)

Figure S1

A

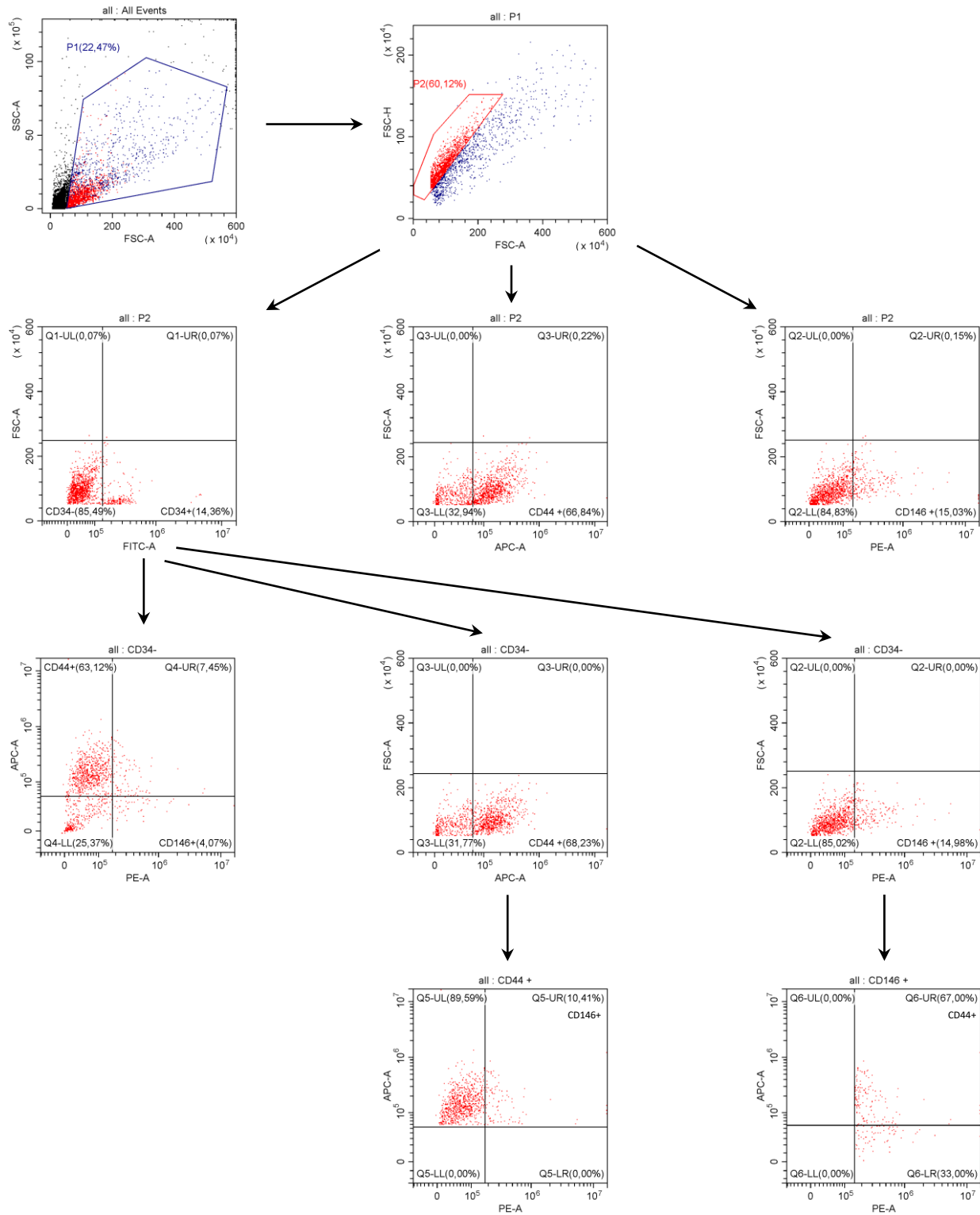

Supplement: Supplementary file 1 — Supplementary file1 (PDF 262 KB) [file 12015_2022_10350_MOESM1_ESM.pdf]
